# Supplementary material for: Transparent near-infrared perovskite light-emitting diodes
Source: Nat Commun. 2020 Aug 24;11:4213. doi: 10.1038/s41467-020-18110-7 (PMC7445238; doi:10.1038/s41467-020-18110-7)
Supplement: Supplementary file 1 — Supplementary Information [file 41467_2020_18110_MOESM1_ESM.pdf]

Supplementary Information

Transparent Near-Infrared Perovskite Light-Emitting Diodes

Chenchao Xie[1,2], Xiaofei Zhao[1], Evon Woan Yuann Ong[1] and Zhi-Kuang Tan[1,2]*

[1] Department of Chemistry, National University of Singapore, 3 Science Drive 3, 117543, Singapore

[2] Solar Energy Research Institute of Singapore, National University of Singapore, 7 Engineering Drive 1, 117574, Singapore

* Correspondence to chmtanz@nus.edu.sg

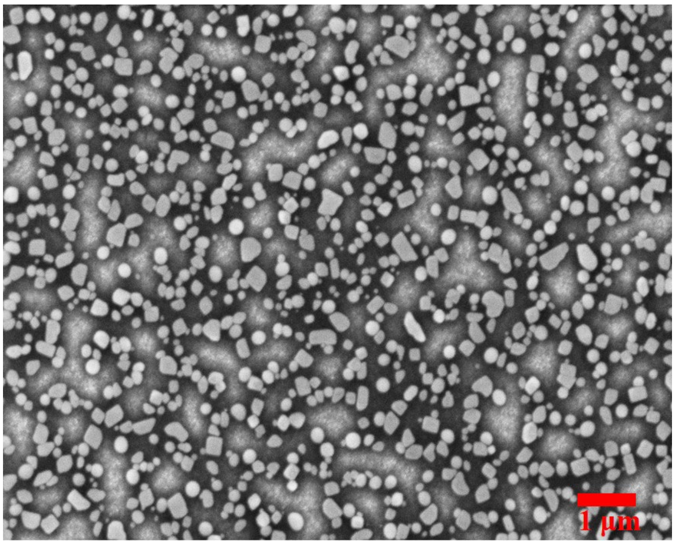

Supplementary Figure 1. Scanning electron microscopy image of the $FAPbI_3$ perovskite layer. Scale bar is 1 µm.

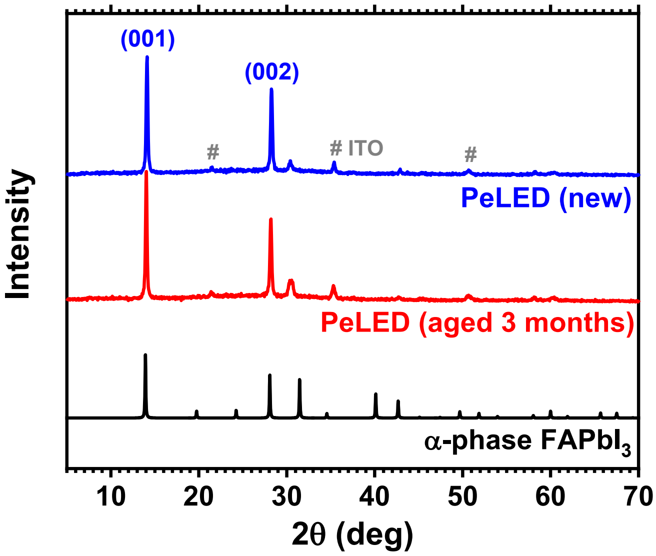

Supplementary Figure 2. X-ray diffraction pattern of $FAPbI_3$ perovskite in PeLED. New and aged devices show negligible differences.

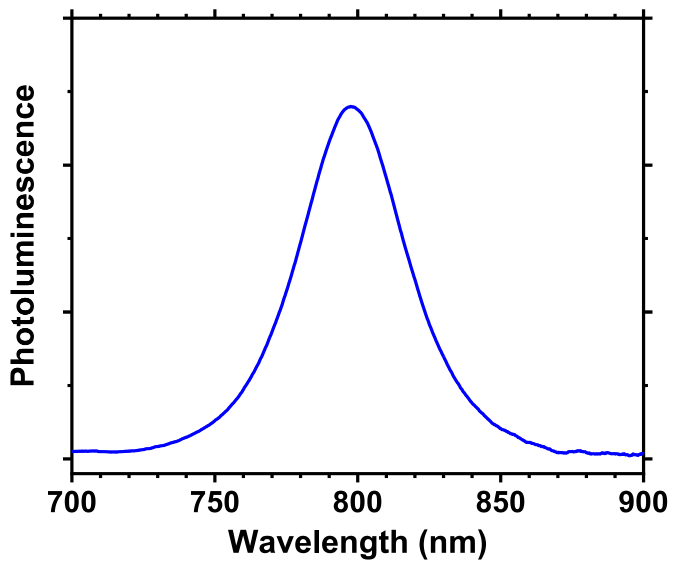

Supplementary Figure 3. Photoluminescence spectrum of $FAPbI_3$ perovskite.

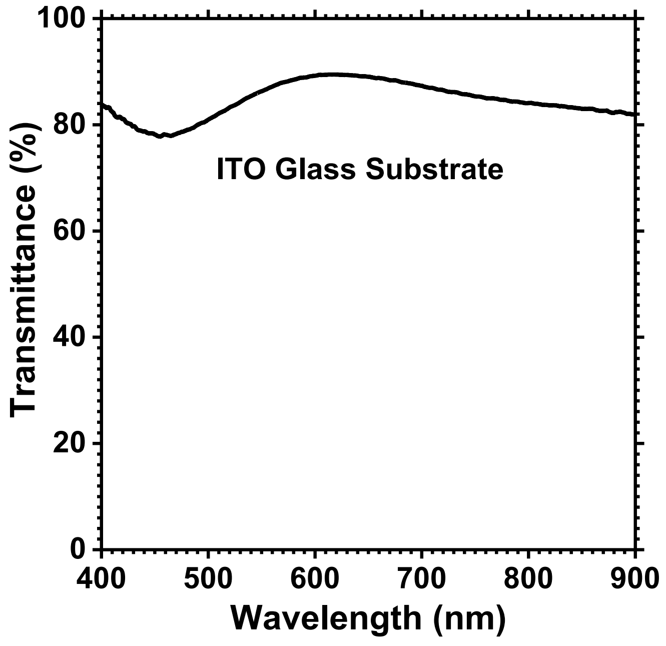

Supplementary Figure 4. Transmittance spectrum of ITO-coated glass substrate.

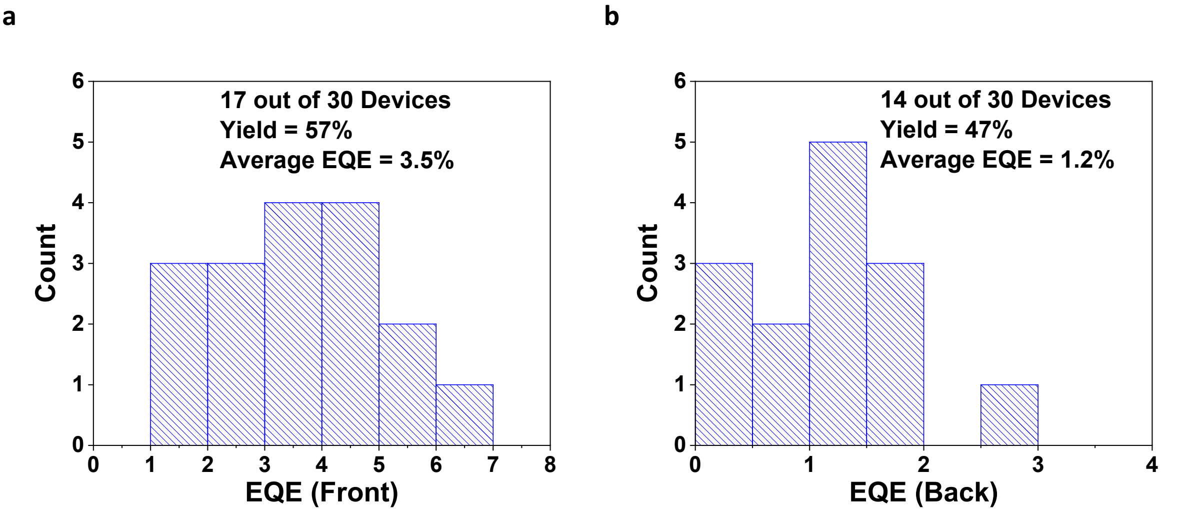

Supplementary Figure 5. Histogram of the maximum external quantum efficiency of the (a) front emission and (b) back emission. The device yield is smaller for the back emission because some devices failed after being driven to 5 V in the front emission measurement.

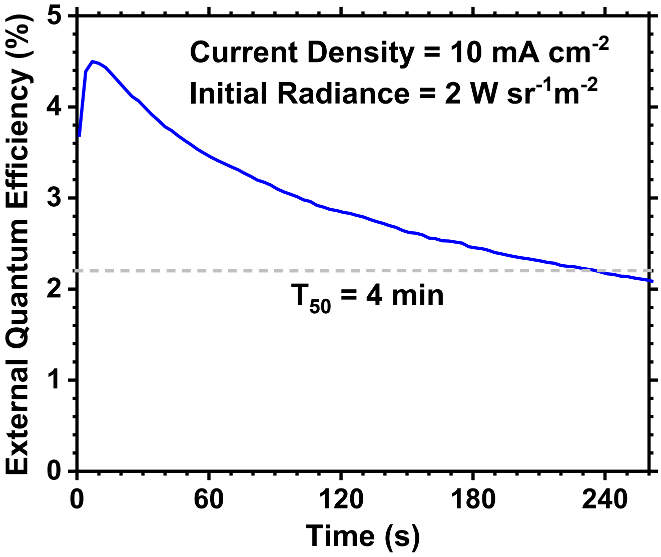

Supplementary Figure 6. Lifetime study of transparent PeLED at a constant current density of 10 mA cm^{-2}.
